# Daily Activity Pattern of Geladas (*Theropithecus gelada,* Ruppell 1835) in Kotu Forest, Northern Ethiopia

**DOI:** 10.1155/2022/7302240

**Published:** 2022-09-26

**Authors:** Degu Abate, Zerihun Girma

**Affiliations:** ^1^Mekdela Amba University, Department of Natural Resource Management, Mekdela Amba, Ethiopia; ^2^Hawassa University, Wondo Genet College of Forestry and Natural Resource, Wondo Gent, Ethiopia

## Abstract

Gelada (*Theropithecus gelada*) is one of the endemic primates of Ethiopia. The ecology of meta populations of geladas outside protected areas is less studied, and their population status is uncertain. As a result, we conducted a study to investigate the daily activity pattern of gelada in Kotu forest and associated grasslands in northern Ethiopia from August 2017 to February 2018 covering both wet and dry seasons. The instantaneous scan sampling method was employed to collect behavioral data. The activity pattern of three selected focal groups of geladas was studied, and predominant behavioral activities were scanned in 15 minutes intervals from dawn 7:00 h to dusk at 18:00 h. Feeding comprised 61.65% of the total scan, followed by moving 18.49%. Feeding activity was more frequent during the dry season (about 65%) than in the wet season (58.20%). On the other hand, moving activity was more frequent during the dry season (about 22%) than in the wet season (about 14%). The daily activity pattern of gelada showed a feeding peak early in the morning and in the late afternoon. The time allocated by geladas for feeding and moving in the study area is higher than other activities. Therefore, there is a need for further in-depth research on diet availability and quality to justify why geladas allocate more time for feeding and moving nexus for conservation interventions.

## 1. Introduction

Wildlife species conservation is based on the understanding of species' life history such as solid data on the daily activity budget of species. Throughout their ranges, primates are threatened mainly due to increased anthropogenic activities that cause habitat degradation, fragmentation, and loss [1]. Due to habitat fragmentation, isolated subpopulations of primates occur over many ranges of primates [1, 2]. This in turn calls for studies in isolated subpopulations elsewhere in their ranges [2, 3]. Moreover, there is a pressing need for such studies when it comes to endemic and endangered species [4–6].

Particularly, the knowledge of the daily activity patterns of primates is important to resource use patterns and selection and assists in developing monitoring strategies for threatened species by providing clues for population count [7–9]. The study of activity patterns is specifically useful for identifying the strategies by which geladas invest time for feeding for survival and reproduction at a given locality [10, 11]. According to the theory of optimality, “the amount of time that an organism spends engaged in various activities depends on the cost of the activity relative to the derived benefits in that organism's habitat” [12]. The daily activity pattern of the primate is directly related to energetic requirements and may vary with seasonal changes in resource availability [13, 14] and reproductive stages [15, 16]. Variations in rainfall and temperature patterns affect food availability and quality, which in turn determine the activity budget of geladas [17, 18]. Therefore, having solid information on the activity pattern of geladas is very crucial to predicting the fitness and survival of geladas at given spatial and temporal scales.

Ethiopia is one of the few countries in the world that possesses unique and characteristic fauna and flora with a high level of endemism [19–23]. Gelada (*Theropithecus gelada*) is one of many endemic mammals of Ethiopia. The endemic gelada was discovered by a German naturalist called Ruppell in 1835 in a few areas of northern Ethiopia. Geladas differ from baboons by the bright patch of skin on their chest [24, 25]. The habitat is characterized by its vicinity to cliffs for sleeping and the use of several different types of relatively treeless and montane grasslands for foraging; habitats that are usually intermixed with bushes, trees, and dens thickets [26–29]. Among primates, geladas are excellent diggers and the only primate which has a specialized graminivorous diet [30, 31].

Primates including endemic species such as gelada are severely threatened by anthropogenic threats such as habitat degradation, fragmentation, loss, poaching, and climate change [6, 32, 33]. Various studies investigated the daily activity patterns of geladas in Ethiopia over protected areas such as Simien Mountain National Park [9, 32, 34, 35] and Guassa Community Conservation Area [33], and some valleys and associated grasslands [5, 14, 28, 36, 37]. However, about 17.62% of Ethiopia's land mass coverage is protected (United Nations Environmental Protection-World Conservation Monitoring Center (UNEPWCMC) [38]. Due to overwhelming habitat fragmentation, metapopulations of geladas exist widely separated apart, inhabiting areas that are inaccessible and unprotected. As a result, metapopulations of geladas over many fragmented areas remain largely unstudied. Moreover, due to environmental and biological variations among habitats of geladas, the diurnal activity pattern could vary among subpopulations. For example, rainfall, temperature, soil, and topographic variations among ranges of geladas could lead to variations in food availability and quality, which in turn lead to activity time budget differences [6, 14, 28]. As a result, this study is curious to answer research questions such as what is the most dominant diurnal activity of geladas at Kotu forest. Is there a seasonal variation among activity patterns of geladas in the Kotu forest and what is the similarity and differences in activity pattern among the present study and other subpopulations of geladas? The study was carried out in one of the unexplored remnant forest patches and associated grassland habitats of gelada found in the northern part of Ethiopia, surrounded by a human-dominated landscape. Particularly, in this study important information on the daily activity patterns of geladas was revealed that inhabit the Kotu Forest and associated grasslands as a proxy for sustainable conservation of the species.

## 2. Methods

### 2.1. Study Area

**Figure 1:**
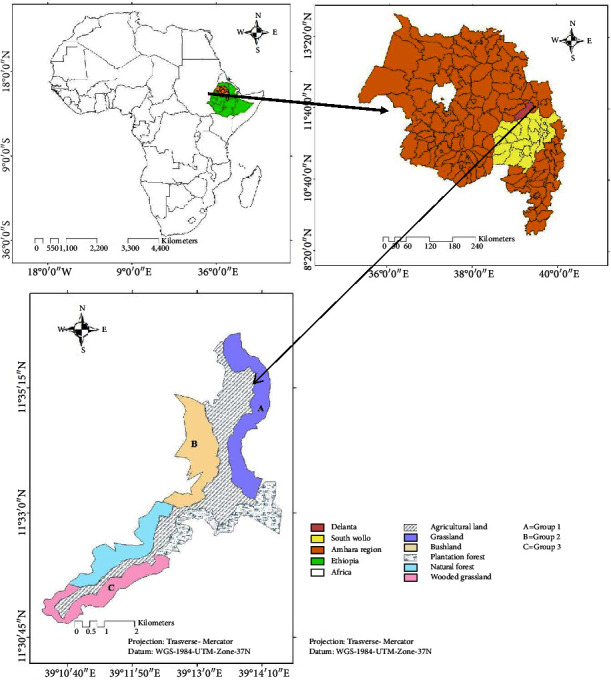
Location map of the study area.

The study was carried out in the Kotu forest located in Northern Ethiopia. It is situated between 11°30′45 ″ to 11°35′45″ N longitude, 39°11′ 50″ to 39°14′10″ *E* latitude (Figure 1). Kotu forest is found in the Amhara Regional State of South Wollo Zonal Administration of Ethiopia (Figure 1). It is located 499 km north of the capital city, Addis Ababa. The total area covered by the forest is 1374 ha. The area is an extensive escarpment of plateaus, hills with small mountainous ridges and gorges, with altitudes ranging from 2771 to 2987 m asl. It has a bimodal rainfall distribution pattern with a long rain season from June to October and a minor rain season from March to May. The dry season in the area occurs from November to February. The annual average maximum and minimum temperature of the study area are 19.8°C and 13°C, respectively [39]. The most predominant flora in the study area includes: *Acacia abysinica, Acacia salingna, Acacia decurrent, Olea europaea, and Juniperous proceara*. Hamadras baboon (*Papio hamadryas),* leopard (*Panthera pardus*), common jackal *(Canis aureus),* spotted hyena *(Crocuta crocuta),* caracal *(Felis carcal),* rock hyrax *(Procavia capensis),* Abyssinian hare *(Lepus habessinicus)*, and klipspringer (*Oreotragus oreotragus*) are among the commonly sighted mammals in the study area [39].

### 2.2. Sampling Design and Data Collection

Before the commencement of the actual data collection, we carried out a reconnaissance survey during the first week of August 2017. The purpose of the reconnaissance survey was to gather basic information about the study area like vegetation (habitat) type and coverage, topographic features, and the range of the species (cliffs and sleeping sites).

The instantaneous scan sampling method was used to collect data on the activity patterns of gelada following [6, 14, 28, 36, 40, 41]. This method requires the detailed observation of multiple group members of the study population. Out of the five dominant habitat types (open grassland, wooded grassland, bushland , natural forest, and plantation forest) that exist in the study area, three dominant habitat types, namely, open grassland, wooded grassland, and bushland habitats were purposively selected for sampling based on ease of sampling and availability of easily recognizable groups of geladas (Figure 1). Three groups, one in each selected habitat, were purposively selected (Figure 1). Group of gelada was defined as a family group that lives, travels, feeds, rests, etc. together and comprises adults (males and females), subadults (males and females), and young, and their number ranges from 13 to 20 individuals. Group one that occurred in the grassland habitat was composed of a total of 20 individuals; 1 adult male, 2 adult females, 3 subadult males, 5 subadult females, and 3 juveniles. Group two that occurred in the bushland habitat was composed of a total of 17 individuals; 1 adult male, 6 adult females, 2 subadult males, 6 subadult females, and 2 juveniles. Group three that inhabited wooded grassland was composed of a total of 13 individuals; 1 adult male, 5 adult females, 1 subadult male, 4 subadult females, and 2 juveniles. One group that permanently inhabits each habitat type was selected based on vigilance effect and topography; vigilance effect and topography structure varied among habitat types. Throughout the study period, three similarly selected groups of geladas were scanned. The main group for scanning was identified using natural marking/scars, size, coat color, and facial features of some distinctive members of each of these groups and also identified by their sleeping sites [14].

Data collection was carried out from August 2017 to February 2018 covering, both the dry and wet seasons. Wet season data collections were conducted from August 2017 to October 2017 and for the dry season from December 2017 to February 2018.

The daily activities of geladas were recorded as they approached targeted groups from 10 to 25 m. Depending on the distance between the observer and the scanned gelada group as well as the topography of the habitat, the behavioral activities were recorded both with naked eyes and aided by 10 × 42 Bushnell binocular [14, 28, 42–44]. To record the behavioral activity pattern of each selected group, 8 consecutive days per month were spent for a total of 7 months covering both dry and wet seasons. The focal group member's predominant behavioral activities were scanned in 15 minutes intervals from dawn at 7:00 h to dusk at 18:00 h [6, 37, 45, 46]. Only activities that were displayed for more than or equal to five seconds were recorded [47]. Behavioral for a single individual was not recorded rather the group was scanned each observation time from left to right to avoid possible biases on eye-catching activities. Group activities were recorded as long as activities were clearly visible with binoculars. When the group was out of sight to clearly observe behavior activities observers moved closer to the group to a clearly observable distance. The behavioral activities were categorized as: feeding, resting, moving, grooming, playing, aggression, sexual activity, and other activities like parental care, vocalization, and defecation [13, 14, 41, 48].

To identify each activity, we followed [41, 49]'s definition of activity categories. When gelada made contact with the food item such as digging and eating the underground food and above-ground food, it was considered a feeding activity. When individuals of gelada are involved in walking, jumping (running) not at the same time feeding, recorded as moving activities. It was considered resting when individuals were sedentary standing, and grooming when geladas use their hands to explore or maintain their body hygiene or the body of other individuals. It was recorded as sexual activity when individuals of gelada participate in copulatory behavior, and as aggression when gelada chase, bit, grab, displace, and threaten another gelada. It was categorized as playing when geladas show friendly chasing, minor hitting, and other vigorous activities.

### 2.3. Data Analysis

We used the statistical package for social science (SPSS) version 21 computer software program to carry out statistical analysis. Statistical tests were two-tailed with 95% of confidence intervals and the rejection level was (*P* ≤ 0.05). To compare seasonal differences (between wet and dry seasons) of each behavioral activity of geladas, we employed the Mann–Whitney *U* test. Data on the number of months per each study season for each behavior was used to compute the Mann–Whitney *U* test. To calculate the overall activity time budget of geladas in the study area the three selected group activities were pulled together and averaged. We calculated the daily activity pattern by dividing the proportion of the number of behavioral records for each activity category by the total number of behavioral activities that were recorded each day. We calculated the monthly activity time budget by averaging the daily values within every four-day record. The grand mean proportion of the monthly budgets provided the overall wet and dry seasons time budgets as well as the overall time budgets during the entire study period [50, 51].

## 3. Results

### 3.1. Activity Pattern

A total of 5661 individual behavioral activity records were obtained during the 1848 scans conducted on 56 study days. The most dominant daily activity pattern of gelada in the Kotu forest was feeding 61.65% (range 52.02%–67.57%, SD ± 5.97%) followed by moving 18.49% (range from 14.43%–25.98%, SD ± 4.96%. Sexual activity was the rarest activity at 1.53% (range from 1.18% to 2.14%, SD ± 0.34%).

Feeding activity was more frequent during the dry (65.09% ± 1.35) season than the wet (58.20% ± 2.32). Likewise, moving was more frequent during the dry season (22.33% ± 1.52) than in the wet season (14.64% ± 0.25). However, most extra activities (resting, grooming, playing, sexual activity, etc.) were more dominant during the wet season than the dry season (Table 1). The nonparametric Mann–Whitney *U* test showed that there was a significant difference between wet and dry seasons in time budget for feeding, resting, grooming, and moving (*P* < 0.05) (Table 1).

During the present behavioral study, the activity time budget of the gelada population varied across months. The percent feeding time budget of gelada baboons during the study period varied, ranging from 52.02% during September to 67.57% during February and moving varied from 14.43% during September to 25.95% during January (Table 2). Likewise, grooming varied ranging from 2.27% in February to 10.4% during August, whereas resting varied, ranging from 1.95% in December to 4.77% in October (Table 2).

**Figure 2:**
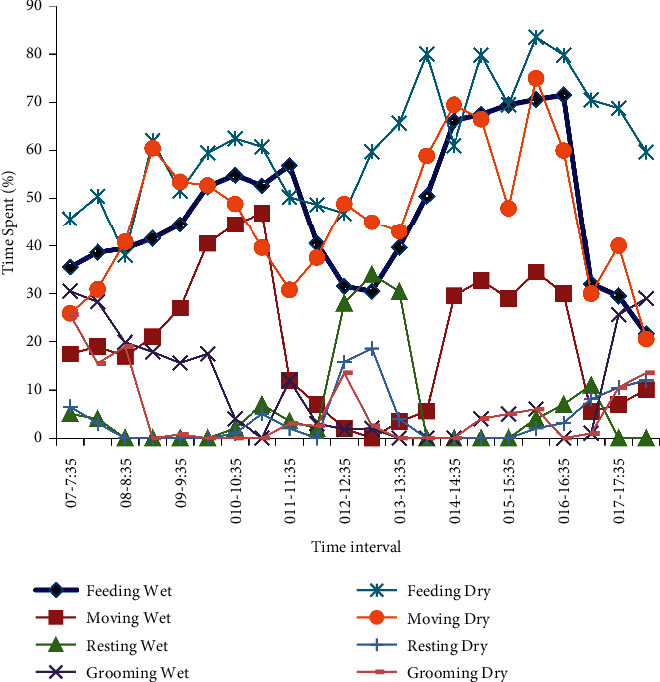
Dominant behaviors diurnal activity pattern of geladas during the wet and dry season in Kotu forest, Ethiopia.

The feeding activity during the wet season increased during the early morning (010:00 to 11:35) hours and declined during the mid-day time (012:00–014:000 h) and peaked again towards early afternoon (014:00–016:35 h) and sharply declined towards the end of the day (after 016:35 h) (Figure 2). Moving was the second most frequent activity and showed four peaks; it peaked during the late morning hours between (09:35–11:00 h) and then declined from (011:00–012:35 h) and peaked again from (013:35 to 16:35) then declined (016:35–017:00 h) (Figure 2). The resting activity was minimal in most morning and afternoon hours, but peaked during noon time (011:35–013:35 h). Grooming was frequent in the early morning hours (07:00 to 08:35 h) and late afternoon hours (016:35–018:00 h), while was rare during the rest hours of the day (Figure 2). Aggression peaked during mid-day (011:00–012:35), the early afternoon hours (014:00–015:00), and towards the end of the day (017:00–017:35). Playing peaked during the morning (07:00–010:00) and late afternoon hours. Sexual activity peaked during the early morning hours (07:00–08:00) and towards the end of the day (016:00–017:00).

During the dry season, geladas spent more time feeding and moving and spent less time on extra activities such as resting, playing grooming, aggression, and sexual activity (Figure 2). During the dry season, feeding occurred throughout the daytime but peaked during the late morning hours (010:00–011:35 h) and early afternoon hours (013:35–015:35 h). Likewise, during the dry season, movement occurred throughout the day time, but peaked during the early morning hours (08:35–09:35 h) and early afternoon hours (014:00–016:00 h) (Figure 2). Grooming peaked during early morning time (07:00–08:35) and occurred for a shorter period of time during the dry season as compared with the wet season (Figure 2). Aggression peaked during the late morning hours (010:35–011:35), at noon (012:00–012:35), the early afternoon hours (014:00–015:00), and towards the end of the day (017:00–018:00). Playing peaked early in the morning (07:00–08:00) and late afternoon hours (015:00–017:00). Sexual activity peaked early the morning (07:00–08:00) and towards the end of the day (017:00–018:00).

## 4. Discussion

The amount of time that primates spend on different activities is directly correlated with energetic requirements [13, 52]. The activities vary with seasons when resource availability is changed in their habitat. There are temperature and rainfall variations between wet and dry seasons in the Kotu forest that determine the resource availability. During the dry season, the average maximum temperature exceeded slightly above 20°C, while during the wet season it dropped to 16 to 17°C. Likewise, the average minimum temperature reached 7 to 8°C during the dry season and dropped to 6 to 8°C during the wet season [39]. On the other hand, the average annual rain falls dropped below 50 mm during the dry season while it peaked at greater than 200 mm [39]. In Kotu forest, geladas spent more than half of (61.65%) of their time feeding over other activity categories. The result is consistent with other geladas activity pattern studies. In Wonchit valley, northeastern Ethiopia [37]; reported feeding as the predominant activity (65.2%) of geladas, and [32]; recorded feeding as the most frequent activity (56.7%) of geladas at Gich Plateau of the Simien Mountains National Park, Northern Ethiopia. Likewise, [28] revealed geladas allocate most of their time (56.12%) to feeding at Debre–Lebanos, central Ethiopia, and [14] reported geladas to spend most of their time feeding (57.19%) at Abogedam church, central Ethiopia.

This study revealed a significant difference between wet and dry seasons in the percentage of gelada time spent on feeding, moving, resting, and grooming activities. According to [3, 28, 32, 53], the quality and availability of food resources cause a seasonal variation in feeding time allocation in geladas. Particularly, [34] as discussed during the dry season the availability and at the same time digestibility of grass species is reduced by half. [32] in their study at Gich Plateau of the Simien Mountains National Park discussed that during the dry season since the availability of green leaves declines and hence geladas have to shift to underground food items which increase processing time. Likewise in the Kotu forest, during field observation, grass blades, grass seeds, herb stems and shoots, and cereal crops were observed to be more frequently consumed during the wet season than the dry season (Personal Observations, 2017). On the other hand, underground roots, bulbs, and fruits were almost exclusively observed to be consumed during the wet season (Personal Observations, 2017). Furthermore, the author discussed that during the dry season, the Gich area experiences near freezing night-time temperatures, which increases the metabolic rate of geladas. To meet the increased energy demand they have to allocate more time for feeding [34]. However, the result contradicts the findings of [36, 37] that reported geladas time spent for feeding did not significantly vary among seasons. The difference in forage quality and quality could also affect reproductive potential. During the wet season, when food resources are abundant, courtship behavior and pregnancy were observed among some adult female geladas, whereas during the early dry season, newborn and parental care behavior increased.

Moving is the second most dominant activity of geladas in Kotu Forest. There was a significant difference in the moving percent time budget between the wet and dry seasons. During the dry season, the time spent moving was higher than in the wet season. Based on field observation, the possible reason could be that during the dry season, sufficient amounts of food and water were not readily available at Kotu forest. During field observation geladas were observed to move long distances in search of green fresh leaves outside the Kotu forest in the dry season, while they did not go far from their cliffs during the wet season (Personal Observations, 2017). Similarly, [3, 6] reported that due to the shortage of food resources during the dry season, geladas spent more time moving. During the wet season, geladas get water from the food that they consume and surface water around their feeding sites. However, during the dry season, grasses dried out and water was not readily available in their foraging sites. So, geladas wander long distances to get water. Similarly, [14, 28, 37] reported that geladas move long distances in search of water during the dry season. The other plausible explanation might be that since the study area is surrounded by agricultural land, crops harvested in the dry season, this provides a good condition for geladas to freely roam in the area. Similarly, [28] revealed that during the dry season around the Debrelibanos areas (central Ethiopia), as crops are harvested, the farmland becomes free. As a result, geladas freely roam within their habitat.

Grooming is one of the social activities of primates and has an important role in maintaining group cohesion and stable relationship [53, 54]. The present study indicated that grooming was higher during the wet season compared to the dry season. During the wet season as compared to the dry season due to high rainfall availability there is greater grass productivity. Consequently, the groups can invest more time in social activities such as grooming and geladas, since they do not travel far for feeding [3].

Due to the high nutritional quality of grasses and forbs, they probably invest relatively less time in feeding and have a better time for social activities such as grooming [6, 14]. Therefore, the possible reason for the increased time allocated for grooming during the wet season than the dry in Kotu forest could be due to the fact that gelada devotes relatively less time to feeding and moving during the wet season than during the dry season. Time spent resting was significantly higher during the wet season than in the dry season in the present study area. In the Kotu forest, geladas could increase the time for resting, reducing the time for feeding in the wet season since food was readily available. Among populations of geladas, the time allocations for resting depend on the time left from feeding [35]. Other similar studies of geladas elsewhere in Ethiopia reported geladas to devote most of their time to feeding and less time to social activities and discussed that during the wet season in most ranges of geladas, quality food is readily available and hence they feed for relatively less time in the wet season and allocate better time for social activities such as resting [6, 28, 33, 37].

## 5. Conclusion and Recommendations

Geladas in the Kotu forest spend more time on feeding and moving and are an important input for the conservation of the endangered species. However, to investigate if the allocation of more time for feeding and moving by geladas is associated with the quality of the habitat of the study area, there is a need for in-depth further study on the analysis of the food availability and quality in the area for more concrete conclusions and conservation interventions. The survival of the gelada baboon depends highly on the planning and implementation of the conservation and management of its habitat resources [25].

## Figures and Tables

**Table 1:** Mean activity time budget (%) and a number of activity events of geladas during the wet and dry seasons in Kotu forest, Ethiopia.

Activity	Activity time budget and number of activity events per season						
	Number of activity events (wet)	Wet activity time budget (%) ± SE	Number of activity events (Dry)	Dry activity time budget (%) ± SE	The overall number of activity events	Total activity time budget (%) mean	*P* value
Feeding	1531.24	58.20 ± 2.32	1972.23	65.09 ± 1.35	3503.47	61.65 ± 1.84	0.001
Moving	385.18	14.64 ± 0.25	676.59	22.33 ± 1.52	1061.77	18.49 ± 1.77	*p* < 0.001
Resting	124.71	4.74 ± 0.31	38.48	1.27 ± 0.06	163.19	3.01 ± 0.19	0.007
Playing	74.98	2.85 ± 0.33	49.99	1.65 ± 0.13	124.97	2.25 ± 0.23	0.152
Grooming	275.93	10.48 ± 0.26	131.5	4.34 ± 0.69	407.43	7.41 ± 0.48	0.003
Aggregation	72.68	2.77 ± 0.28	47.9	1.58 ± 0.33	120.58	2.18 ± 0.31	0.347
Sexual activity	43.15	1.64 ± 0.17	42.72	1.41 ± 0.53	85.87	1.53 ± 0.35	0.229
Other activities	123.13	4.68 ± 0.38	70.59	2.33 ± 0.04	193.72	3.51 ± 0.21	0.453
Total	**2631**	**100%**	**3030**	100%	**5661**	**100%**	

**Table 2:** Monthly activity time budget (%) and a number of activity events of geladas among different activity behavioral categories (Mean ± SE) in Kotu forest, Ethiopia.

Activity type	Monthly activity time budget and number of activity events															
	No. of activity events	Aug%	No. of activity events	Sep%	No of activity events	Oct%	No. of activity events	Nov%	No. of activity events	Dec%	No. of activity events	Jan%	No of activity events	Feb%	Total no. of activity events	Mean ± (SE)
Feeding	358.32	55.43	336.28	52.02	433.7	67.09	402.95	62.33	621.2	61.31	666.39	65.77	684.63	67.57	3503.47	61.65 ± 2.25
Moving	93.16	14.45	93.04	14.43	102.84	15.95	96.13	14.91	240.58	24.77	252.06	25.95	183.96	18.94	1061.77	18.49 ± 1.88
Resting	26.82	3.26	34.47	4.19	39.26	4.77	24.16	2.94	12.74	1.95	12.84	1.96	12.94	1.98	163.19	3.01 ± 0.43
Playing	23.28	2.95	7.89	1.00	17.92	2.27	25.88	3.28	8.21	1.03	22.72	2.85	19.06	2.39	124.97	2.25 ± 0.34
Grooming	73.36	10.4	64.75	9.18	65.24	9.25	72.58	10.29	56.94	5.53	51.18	4.97	23.38	2.27	407.43	7.41 ± 1.19
Aggregation	20.10	2.65	14.34	1.89	23.14	3.05	15.09	1.99	18.82	2.22	13.06	1.54	16.02	1.89	120.58	2.18 ± 0.19
Sexual activity	10.03	1.20	9.87	1.18	11.54	1.38	11.71	1.4	16.53	2.14	13.75	1.78	12.44	1.61	85.87	1.53 ± 0.13
Other activities	41.17	6.62	20.27	3.26	35.14	5.65	26.55	4.27	23.34	1.62	20.31	1.41	26.94	1.87	193.72	3.51 ± 0.78

## Data Availability

Data that support the findings of the research will be made available upon request.
